# Tangzhiqing Granules Alleviate Podocyte Epithelial-Mesenchymal Transition in Kidney of Diabetic Rats

**DOI:** 10.1155/2017/1479136

**Published:** 2017-01-10

**Authors:** Haiyan Xu, Xu Wang, Mingming Liu, Xueyuan He

**Affiliations:** ^1^The First Clinical Medical College, Nanjing University of Chinese Medicine, Nanjing, Jiangsu 210023, China; ^2^Xuzhou Medical University, Xuzhou, Jiangsu 221004, China

## Abstract

This study discussed the effect of Tangzhiqing granules on podocyte epithelial-mesenchymal transition in kidney of diabetic rats. The diabetic rats were divided randomly into five groups: DM group treated with vehicle, Tangzhiqing granules low-dose treatment group, Tangzhiqing granules middle-dose treatment group, and Tangzhiqing granules high-dose treatment group. Eight Wistar rats used as control group were given saline solution. The intervention was all intragastric administration for 8 weeks. At the end of the 8 weeks, biochemical parameters and kidney weight/body weight ratio were measured. The kidney tissues were observed under light microscope and transmission electron microscopy. To search for the underlying mechanism, we examined the epithelial-to-mesenchymal transition (EMT) related molecular markers and TGF-*β*/smad signaling pathway key proteins expression. The results showed that Tangzhiqing granules relieved the structural damage and functional changes of diabetic kidneys. Kidney podocyte EMT related molecular markers nephrin and CD2AP expression were increased, when desmin and *α*-SMA levels were decreased by Tangzhiqing granules in diabetic rats. Further TGF-*β*/smad signaling pathway key proteins TGF-*β*1 and p-smad2/3 levels were decreased in diabetic rats after treatment with Tangzhiqing granules. These findings suggest that Tangzhiqing granules may protect the podocytes of diabetic nephropathy rats via alleviating podocyte EMT and likely activating TGF*β*/smad signaling pathway.

## 1. Introduction

Diabetic nephropathy is one of the most common microvascular complications of diabetes, which eventually develops to end-stage renal disease. Studies in vivo and in vitro found that glomerular podocytes exposed to high glucose concentration could appear as phenotype transformation, which caused podocytes structure and function injury and proteinuria to be aggravated [1]. Renal biopsy results of diabetic nephropathy patients also confirmed the existence of podocyte phenotype transformation [2, 3]. Podocyte phenotype transformation mainly refers to the pathological state of podocyte epithelial-mesenchymal transition (EMT), which results in loss of podocyte epithelium specificity and damage to podocyte function [4]. TGF-*β*/smad signaling pathway is one of the most important signal pathways which mediate kidney podocytes epithelial-mesenchymal transition.

Chinese medicine compound can effectively improve the clinical symptoms of diabetic nephropathy patients and obviously reduce urinary protein through multifactorial and multitarget ways. Previous studies have shown that Tangzhiqing granules which were the experience prescription of Chinese medicine master Zhou Zhongying could relieve 24 hours urinary protein, improve the endogenous creatinine clearance, and protect the kidney function of diabetic nephropathy rats. This study was designed to observe the influence of Tangzhiqing granules on podocytes EMT, to discuss the possible mechanism of Tangzhiqing granules protecting glomerular podocytes.

## 2. Materials and Methods

### 2.1. Experiment Animals

Sixty male Wistar rats (180–220 g) aged 8 weeks were purchased from the Experimental Animal Center of Nanjing university of Chinese medicine. Every three rats were fed in one cage under normal conditions with a 12 h light/dark cycle at 16–26°C and relative humidity of 40%–60%. Animals were all treated strictly according to the requirements of the Ethics Committee of Nanjing university of Chinese medicine. Every effort was made to minimize the suffering of experimental animals.

### 2.2. Tangzhiqing Granules

Tangzhiqing granules was prepared by the manufacturing laboratory of Jiangsu Provincial Hospital of Traditional Chinese Medicine. Tangzhiqing granules consist of* rhizoma polygonati*, matrimony vine, herba lycopi*, Bombyx batryticatus*, and* euonymus alatus*.

### 2.3. Reagents

Streptozotocin (STZ) was obtained from Sigma Chemical Co. (MO, USA). Blood glucose kit, serum creatinine kit, blood urea nitrogen kit, cholesterol kit, triglycerides kit, and urinary albumin kit were purchased from Jiancheng Technology Co. (Nanjing, China). The antibodies against nephrin, *α*-SMA, *β*-actin, and goat anti-rabbit IgG-HRP were purchased from Abcam, Inc. (Cambridge, UK). Desmin antibody was purchased from Cell Signaling Technology (MA, USA). CD2 associated protein antibody (CD2AP) was purchased from Signalway Antibody LLC (MD, USA). TGF-*β*1 and p-smad2/3 antibodies were bought from Bioworld Technology, Inc. (MN, USA).

### 2.4. Experimental Design

High-fat diet combined with low-dose STZ intraperitoneal injection were used to establish diabetic models as mentioned previously [5, 6]. Briefly, all Wistar rats were fed with high-fat diet for 6 weeks. Then a single intraperitoneal (i.p.) injection of a low dose of STZ (30 mg/kg, made freshly in citrate buffer, pH 4.5) was administered. Blood glucose level was measured 72 h after the injection. The rats were excluded if their blood glucose was less than 16.7 mmol/L. The diabetic rat models were successfully established when blood glucose had been stable for more than 5 days. These rats were fed a high-fat diet but not given any hypoglycemic agent throughout the experimental period. Eight normal Wistar rats used as control group were given intragastric administration of saline solution. The diabetic rats were divided randomly into four groups: DM group (*n* = 8) treated with vehicle alone, TZQ1 group (*n* = 8) treated with Tangzhiqing granules 3 mg/kg, TZQ2 group (*n* = 8) treated with Tangzhiqing granules 6 mg/kg, and TZQ3 group (*n* = 8) treated with Tangzhiqing granules 12 mg/kg. The intervention was all intragastric administration for 8 weeks. At the end of the 8 weeks, each rat was put into metabolic cage to collect 24 h urine for the measurement of urinary protein (24 hUPro). After 8 weeks of treatment, the rats were intraperitoneally anesthetized and peripheral blood was collected to detect blood glucose (BG), serum creatinine (Scr), blood urea nitrogen (BUN), cholesterol (CHOL), and triglyceride (TG). The ratio of right kidney weight/body weight (KI) for each rat was calculated. Then the kidneys were isolated, cleaned, and resected. All the kidneys of each rat were cut in a coronal plane. A quarter dorsal part of the right kidney was used for observation under light microscope. Another quarter dorsal part of the right renal cortex was cut into small pieces (1 mm^3^) for transmission electron microscopy observation. One-half of dorsal part in the right renal cortex was fixed in 4% paraformaldehyde for immunohistochemistry test. The remaining dorsal parts of the renal cortical tissues were stored at −80°C for further researches.

### 2.5. Measurement of Biochemical Parameters

Serum creatinine, blood urea nitrogen, cholesterol, triglycerides, and urinary albumin were determined using Beckman automatic biochemical analyzer. Blood sugar was tested by the Bayer blood glucose meter.

### 2.6. Morphologic Analysis

Parts of renal tissue were fixed in 4% paraformaldehyde liquid more than 24 h and then embedded in paraffin for study by light microscopy. Two kidney sections about 3 *μ*m thickness from each rat were stained with hematoxylin-eosin reagent. Kidney pathological morphological changes were detected under microscope and magnified 200 times to collect images. Ten images were randomly selected from the two slides of each rat and evaluated by two investigators who were blinded to the origin of the slides. The glomerular volume was calculated using the following formula: glomerular volume = 4*πr*^3^/3. For transmission electron microscopy examination, 1 mm^3^ renal cortex was fixed in 2.5% glutaraldehyde solution, rinsed by phosphate buffer, fixed in 10% osmic acid, and then embedded in epon812 according to standard procedures. About 70 nm ultrathin sections were cut and stained with both uranyl acetate and lead citrate. Then glomerular morphology, especially the podocyte changes, was observed using an JEM-1011 transmission electron microscope (Japanese electronics co., Tokyo, Japan).

### 2.7. Real-Time Polymerase Chain Reaction

Total RNA was isolated from the renal cortex tissues using Trizol reagent (Invitrogen, Carlsbad, CA, USA) following the manufacturer's instructions. The extracted RNA was reverse transcribed to first strand cDNA using a First Strand cDNA Synthesis Kit (Toyobo CO., LTD., Shanghai, Japan). Synthesised cDNA was amplified by a standard real-time polymerase chain reaction (PCR) protocol using SYBR Green PCR Master Mix (Roche, Basel, Switzerland) to measure the expression levels of the target genes. The primer sets for nephrin, CD2AP, desmin, *α*-SMA, TGF-*β*1, and *β*-actin were purchased from Shanghai Sangon Biological Engineering Technology (Shanghai, China). The primers used were as follows: nephrin, forward 5′-TTCTTGTTCTCCGATTGT-3′ and reverse 5′-CCCAGTCAGCGTGAAGGTAG-3′; CD2AP, Forward 5′-CCATCCTTCTTCCTG TAG-3′ and Reverse 5′-TGACTATATTGTGGAGTATGA-3′; desmin, Forward 5′-GCATCGTTGTTCTTATTGG-3′ and Reverse 5′-TCAGTAT GAGACCATTGC-3′; *α*-SMA, forward 5′-CACCTATAACAG CATCAT-3′ and reverse 5′-GAGGAGCAATAATCTT GA-3′; TGF-*β*1, forward 5′-ACAGTTGACTTGAATCTC-3′ and reverse 5′-AACGCAATCTATGACA AA-3′; *β*-actin, forward 5′-GGCTGTATTCCCCTCC ATCG-3′ and reverse 5′-CCAGTTGGTAACAATGCCATGT-3′. A volume of 20 *μ*L reaction system included SYBR Green PCR Master Mix, forward primers, reverse primers, cDNA, and DEPC water. The real-time PCR conditions were as follows: 10 min initial denaturation at 95°C, then 40 cycles denaturation at 95°C for 15 s, and annealing at 60°C for 1 min. The relative expression level of the target mRNAs was normalized over the *β*-actin mRNA level in the same sample and computed using the 2^−ΔΔCt^ method.

### 2.8. Western Blotting

Total protein of the renal cortex tissues was extracted and the concentration was measured using a BCA kit. The proteins (20 *μ*g) were separated by SDS-PAGE and transferred onto polyvinylidene fluoride (PVDF) membranes (Bio-Rad Laboratories, Inc.). The PVDF membranes were blocked with 5% nonfat milk in TBST for two hours at room temperature and then incubated with primary antibody at 4°C overnight. After further incubation with anti-rabbit IgG conjugated to horseradish peroxidase, the immunoreactivity in the bands was observed using the ECL substrate according to the manufacturer's protocol. Beta-actin antibody was used as a control. Densitometric analysis was carried out with ImageJ software (National Institutes of Health).

### 2.9. Immunohistochemistry

The kidney tissues collected at the end of the 8 weeks were fixed with 4% paraformaldehyde for histological analysis according to the standard protocols. Renal sections (4 *μ*m thickness) were deparaffinized in xylene and rehydrated in graded ethanol. Then the sections were treated with normal goat serum to block nonspecific staining. The kidney slides were incubated with the primary antibody overnight at 4°C. For immunohistochemical negative controls the kidney sections were incubated with rat normal serum.

### 2.10. Statistical Analysis

Statistical analysis was performed using IBM SPSS statistics 20.0 and all results were expressed as mean ± SD. Comparisons among groups were performed using one-way ANOVA. A value of *P* < 0.05 was considered to indicate a statistically significant difference.

## 3. Results

### 3.1. Physiological and Biochemical Indicators

After 8 weeks of treatment, we measured the levels of BG, BUN, Scr, 24 hUpro, CHOL, TG, Weight, and KI (shown in Table 1). The BG, BUN, Scr, Upro, CHOL, TG, and KI levels were significantly increased, when the weight decreased in DM group compared with control group (all *P* < 0.05). BG, BUN, Scr, Upro, KI, and CHOL levels in both TZQ2 and TZQ3 groups were significantly decreased, and the weight were increased, compared with DM group (all *P* < 0.05). And the CHOL levels were also decreased in the TZQ1 group. The TG level was decreased only in TZQ3 group. There were no obvious statistical differences between TZQ2 and TZQ3 groups about all the indicators except for the BG levels.

### 3.2. Effects of Tangzhiqing Granules on Renal Tissue Morphology

**Figure 1:**
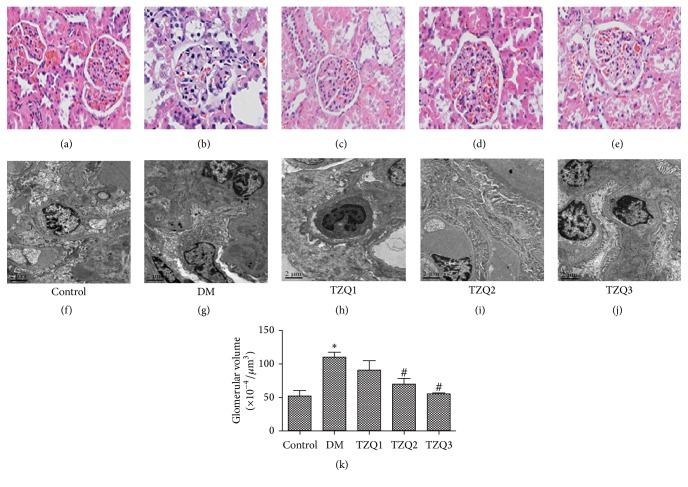
Renal histopathology was observed after 8 weeks of treatment in five groups under light microscopy; HE stained; ×200 magnification (a–e). Ultrastructural features of renal cortex tissues were observed under transmission electron microscopy; ×5000 magnification (f–j). (a, f) Control group; (b, g) DM group; (c, h) TZQ1 group; (d, i) TZQ2 group; (e, j) TZQ3 group; (k) glomerular volume. The data were expressed as mean ± SD; ^*∗*^*P* < 0.05 versus control group; ^#^*P* < 0.05 compared to DM group.

The renal morphology of rats in five groups after 8 weeks of treatment was observed under light microscopy and transmission electron microscopy (Figure 1). The kidney structure changes under light microscope in each group were shown in Figures 1(a)–1(e). In DM group, glomerular volume increasing, mesangial matrix expansion, capillary wall thickening, and a mass of renal tubular epithelial cells vacuoles degeneration were all considered consistent with the pathology of diabetic nephropathy (Figures 1(b) and 1(k)). However, all of the pathological changes above were ameliorated after being treated with Tangzhiqing granules in both TZQ2 and TZQ3 groups (Figures 1(d), 1(e), and 1(k)). The renal tissue morphology in TZQ1 group was almost the same as in the DM group. Then glomerular podocytes were examined by transmission electron microscopy to investigate the effects of Tangzhiqing granules on renal ultramicrostructure (Figures 1(f), 1(g), 1(h), 1(i), and 1(j)). Ultrastructure observation of renal cortical tissue in control group showed normal glomerular basement membrane (GBM) without foot process fusion (Figure 1(f)). But in DM group, some foot processes of podocytes were fused, even completely ruined and vanished. Meanwhile, the GBM became thicker and mesangial matrix was expanded in DM group compared to the control group (Figure 1(g)). However, both the podocyte injury and glomerular basement membrane thickness (GBMT) were alleviated by Tangzhiqing granules in TZQ2 and TZQ3 groups (Figures 1(i) and 1(j)). But there was no obvious improvement on the pathological changes in TZQ1 group. All these results showed that Tangzhiqing granules relieved the kidney injury of diabetic rats both under light microscopy and transmission electron microscopy.

### 3.3. Regulation of Tangzhiqing Granules on Expression of Epithelial-to-Mesenchymal Transition Related Molecular Markers Nephrin, CD2AP, Desmin, and *α*-SMA in Renal Cortex Tissues of Rats

**Figure 2:**
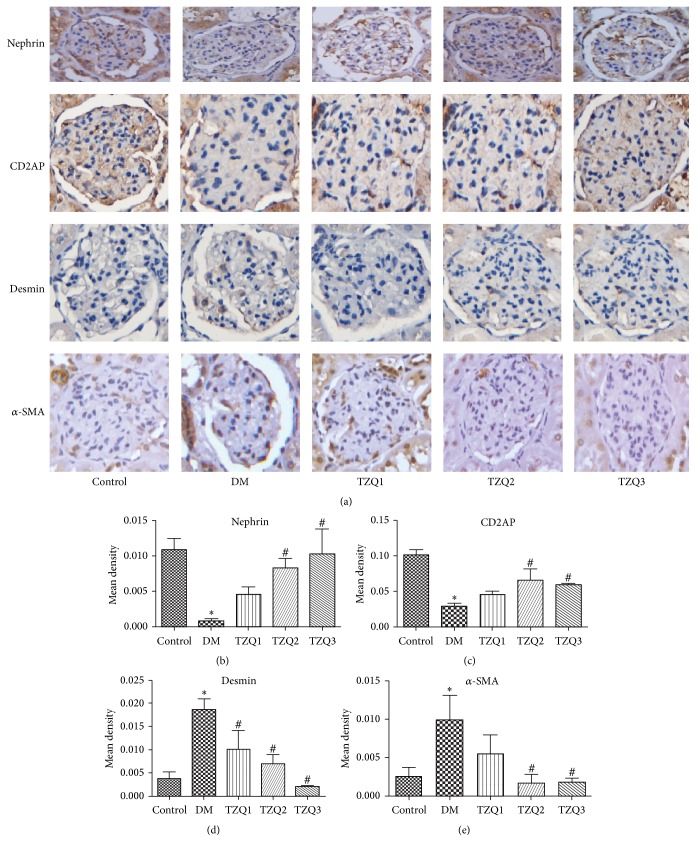
Immunohistochemical staining for nephrin, CD2AP, desmin, and *α*-SMA accumulation in renal cortical tissues of the five groups. (a) Immunohistochemical staining for nephrin, CD2AP, desmin, and *α*-SMA; (b) mean density for nephrin; (c) mean density for CD2AP; (d) mean density for desmin; (e) mean density for *α*-SMA. The data were expressed as mean ± SD; ^*∗*^*P* < 0.05 versus control group; ^#^*P* < 0.05 compared to DM group.

**Figure 3:**
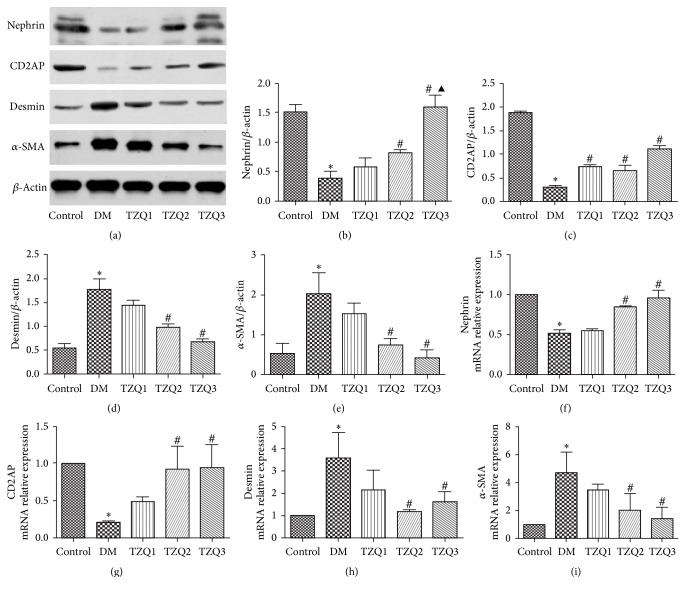
Effect of Tangzhiqing granules on expression of nephrin, CD2AP, desmin, and *α*-SMA protein and mRNA in renal cortex tissues in five groups. Nephrin, CD2AP, desmin, and *α*-SMA protein expression levels were analyzed by western blotting (a). Relative protein expression levels of nephrin, CD2AP, desmin, and *α*-SMA were shown in (b–e). Data were presented as mean ± SD and normalized to *β*-actin protein expression. The mRNA expression levels of nephrin, CD2AP, desmin, and *α*-SMA were assessed by real-time PCR (f–i). The data were expressed as mean ± SD and normalized to *β*-actin mRNA expression. ^*∗*^*P* < 0.05 versus control group; ^#^*P* < 0.05 compared to DM group; ^▲^*P* < 0.05 compared to TZQ2 group.

After 8 weeks of treatment, the epithelial-to-mesenchymal transition related molecular markers nephrin, CD2AP, desmin, and *α*-SMA protein and mRNA expression levels were measured (Figures 2 and 3). Immunohistochemical staining for nephrin, CD2AP, desmin, and *α*-SMA accumulation in renal tissues of the five groups was shown in Figure 2(a). Nephrin and CD2AP protein expression was decreased significantly in DM group when compared to the control group. Treatment with Tangzhiqing granules in both TZQ2 and TZQ3 groups significantly restored the decreased protein (Figures 2(a), 2(b), and 2(c)). But desmin and *α*-SMA protein expression was increased significantly in DM group. The increased protein was restored in the TZQ2 and TZQ3 groups (Figures 2(a), 2(d), and 2(e)).

Nephrin, CD2AP, desmin, and *α*-SMA protein expression levels analyzed by western blotting and mRNA expression levels assessed by real-time PCR were all shown in Figure 3. The protein and mRNA levels of nephrin and CD2AP were markedly decreased in DM group compared with the control group, while in both TZQ2 and TZQ3 groups they were increased compared with DM group (Figures 3(a), 3(b), 3(c), 3(f), and 3(g)). But desmin and *α*-SMA protein expression levels were significantly increased in DM group compared to the control group and were decreased in both TZQ2 and TZQ3 groups (Figures 3(a), 3(d), and 3(e)). In TZQ2 and TZQ3 group, the desmin and *α*-SMA mRNA expression levels were the same trend as the protein levels (Figures 3(h) and 3(i)). For nephrin, CD2AP, desmin, and *α*-SMA levels, there were no significant differences between the TZQ2 and TZQ3 group (Figures 2 and 3).

### 3.4. Effect of Tangzhiqing Granules on TGF-*β*/smad Signaling Pathway in Renal Cortex Tissues of Rats

**Figure 4:**
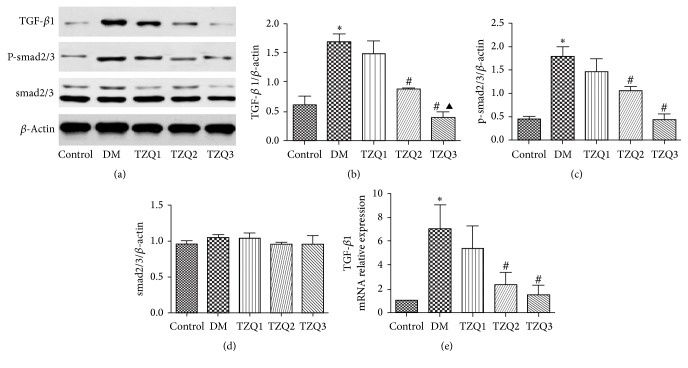
Effect of Tangzhiqing granules on TGF-*β*1, p-smad2/3, and smad2/3 expression levels in renal cortex tissues. Protein expression levels of TGF-*β*1, p-smad2/3, and smad2/3 were analyzed by western blotting (a). Relative protein expression levels of TGF-*β*1, p-smad2/3, and smad2/3 were shown in (b), (c), and (d). Data were presented as mean ± SD and normalized to *β*-actin protein expression. The mRNA expression levels of TGF-*β*1 were assessed by real-time PCR (e). The data were expressed as mean ± SD and normalized to *β*-actin mRNA expression. ^*∗*^*P* < 0.05 versus control group; ^#^*P* < 0.05 compared to DM group; ^▲^*P* < 0.05, compared to TZQ2 group.

After 8 weeks of treatment, TGF-*β*/smad signaling pathway related proteins expression levels were measured (Figure 4). The protein and mRNA levels of TGF-*β*1 were markedly increased in DM groups compared with the control group, while in TZQ2 and TZQ3 groups they significantly decreased compared with DM group (Figures 4(a), 4(b), and 4(e)). P-smad2/3 protein expression levels were significantly increased in DM group and decreased in the TZQ2 and TZQ3 groups (Figures 4(a) and 4(c)). But the protein expression level of smad2/3 had no significant differences in the five groups (Figures 4(a) and 4(d)). For both TGF-*β*1 and P-smad2/3 levels, there were no significant differences between the TZQ2 and TZQ3 groups (Figures 4(a), 4(b), 4(c), and 4(e)).

## 4. Discussion

Diabetic nephropathy is one of the most serious complications of diabetes mellitus, eventually resulting in dialysis or kidney replacement therapy for patients [7]. Traditional Chinese medicine has been used for the treatment of diabetes and diabetic nephropathy for thousands of years, getting more and more attention [8]. Tangzhiqing granules which is the empirical formula of Chinese medicine master Zhou Zhongying, contain five Chinese herbs, such as* rhizoma polygonati*, matrimony vine, herba lycopi,* Bombyx batryticatus,* and* euonymus alatus*. The earlier clinical studies in our team have shown that Tangzhiqing granules had significant effect on diabetic nephropathy. Animal experiments have shown that Tangzhiqing granules could improve the blood glucose, blood lipid, and renal function of diabetic rats. This study aimed to discuss the mechanisms of Tangzhiqing granules reducing urinary protein and improving kidney function.

After 8 weeks of treatment with Tangzhiqing granules in both TZQ2 and TZQ3 groups, BG, BUN, Scr, 24 hUpro, and KI levels were significantly decreased compared with the DM group. The CHOL levels in TZQ1, TZQ2, and TZQ3 groups were all lower than in the DM group. The TG levels only in TZQ3 group were decreased compared to DM group. The results showed that Tangzhiqing granules could regulate glucolipid metabolic disorders and relieve diabetic nephropathy. Under light microscope, pathological changes of diabetic nephropathy were obviously ameliorated after being treated with Tangzhiqing granules in TZQ2 and TZQ3 groups. The renal ultramicrostructure observation manifested that both the podocyte injury and glomerular basement membrane thickness were alleviated by the treatment of Tangzhiqing granules. All these results showed that Tangzhiqing granules could relieve both the structural damage and functional changes of diabetic nephropathy rats significantly in the two Tangzhiqing granules groups.

Experiments in vivo and in vitro have found that high glucose could lead to epithelial-to-mesenchymal transition (EMT) in podocytes. This kind of phenotype change resulted in further structure and function damage in podocytes, which eventually caused the proteinuria occurrence and development of DM [1, 9]. Pathological factors such as hyperglycemia and oxidative stress have been confirmed to induce podocytes EMT [10]. Researches have shown that inhibition of podocytes EMT could attenuate urinary protein and renal injury of diabetic nephropathy [11]. Podocytes EMT could be reflected by the changes of protein markers, such as reduced expression of epithelial marker proteins and increased expression of mesenchymal cell marker proteins [12]. Epithelial marker proteins include nephrin and CD2AP, while mesenchymal cell marker proteins include desmin and *α*-SMA. Nephrin, one of transmembrane proteins, is located in the glomerular slit diaphragm, playing an important role in the development and function maintaining of the glomerular filtration barrier. Decrease of nephrin protein expression affects the cell signal transduction, which results in further damage in podocyte structure, causes foot process fusion and even loss and development of proteinuria [13, 14]. CD2 related protein (CD2AP) is also a glomerular slit diaphragm molecule, associated with urinary protein. It can mediate interactions between protein molecules and signal transduction [15]. Through connecting with nephrin protein, CD2AP links glomeruli podocyte slit diaphragm to the cytoskeleton in the foot processes, stabilizes the slit diaphragm, and participates in maintaining podocytes morphology and function [16]. Desmin is cytoskeleton intermediate filament protein, which is less expressed in the kidney podocytes under normal condition. Studies found that, when podocytes were damaged, changes of cytoskeleton arrangement could lead desmin expression to be increased significantly and podocyte phenotype to be changed. So desmin is often used as the marker protein of podocyte damage [17, 18]. Alpha smooth muscle actin (*α*-SMA) is the marker protein of myofibroblasts, normally only expressed in renal blood vessels. Researches showed that damage by pathological factors such as hyperglycemia and renal inherent cells could appear as EMT and expression of *α*-SMA protein. It is often used as a marker protein of mesenchymal cells in studies on glomerular podocytes EMT [19]. In this experiment, the protein and mRNA levels of nephrin and CD2AP which were epithelial cells marker proteins were markedly decreased in DM group and increased by Tangzhiqing granules in TZQ2 and TZQ3 groups. But the mesenchymal cell marker proteins desmin and *α*-SMA expression levels were significantly increased in DM group and were decreased by Tangzhiqing granules in TZQ2 and TZQ3 groups. Our study showed that Tangzhiqing granules improved the podocyte phenotypic changes in diabetic rats, but the number of podocytes could decrease under high glucose conditions. Loss of podocytes is usually accompanied with decreased podocyte marker protein, such as nephrin and CD2AP. So further experiment will be needed to explore whether the loss of nephrin and CD2AP is related to podocyte loss. Staining and quantification of Wilms' tumor 1 protein which is a specificity core protein in kidney podocytes will be supplemented.

TGF-*β* is one of the most important regulatory factors in podocytes EMT. TGF*β*/smad signaling pathway plays an important role in mediating renal fibrosis. Under the action of TGF*β*1, transmembrane protein TGF-*β* type II receptor and type I receptor form a closely integrated complex on the cell membrane. Then the complex activates intracellular smad2 and smad3. Dissociated from the receptors, phosphorylated smad2/3 complex combines with smad4 and then enters the nucleus. The new complexes interact with cis-acting elements in the nucleus to adjust the EMT related gene expression [20]. To investigate the mechanism of Tangzhiqing granules reversing kidney podocytes EMT, we tested the TGF-*β*/smad signaling pathway key molecular TGF-*β*1 and p-smad2/3 expression levels. The protein and mRNA levels of TGF-*β*1 were markedly increased in DM group and decreased by Tangzhiqing granules with dosage of 6 mg/kg and 12 mg/kg. Increased p-smad2/3 protein levels were also significantly decreased by Tangzhiqing granules, but the levels of smad2/3 had no significant differences in the five groups. These results showed that Tangzhiqing granules could affect the expression of TGF-*β*1 and phosphorylated smad2/3 but had no effect on the nonphosphorylated smad2/3 levels of the renal cortex in diabetic rats. TGF-*β*1 and p-smad2/3 levels had no statistical difference between TZQ2 and TZQ3 groups. The effect of Tangzhiqing granules adjusting TGF-*β*/smad signaling pathway was not in a dose-dependent manner.

According to the literatures, the constituents of Tangzhiqing granules could improve diabetes and its complications [21–25]. The water-extraction of herba lycopi and radix astragali could improve the clinical symptoms for the DN rats which were caused by STZ. The randomized and controlled clinical trial indicated remarkable protective effects of* Lycium barbarum* polysaccharide in patients with type 2 diabetes. Serum glucose decreased significantly and insulinogenic index increased and high-density lipoprotein levels also increased after 3 months administration with* Lycium barbarum* polysaccharide. The water extract of* rhizoma polygonati* odorati significantly decreased hyperglycemia and significantly prevented hypertriglyceridemia in diabetic rats.* Euonymus alatus* ethyl acetate fraction could significantly decrease glycosylated serum protein, triglyceride, and total cholesterol in diabetic mice. Additional studies also showed it had beneficial effects on histopathological of pancreatic islets in diabetic mice. Experimental studies found that* Bombyx batryticatus* especially in powder had hypoglycemic effect on diabetic rats. This study showed that Tangzhiqing granules could regulate glucolipid metabolic disorders. High glucose and hyperlipid play an important role in kidney podocytes EMT. So we could not exclude that Tangzhiqing granules improving DM rats podocyte EMT is related to improving glucolipid metabolic disorders. Further experiments in vitro will be needed to verify the findings. But our research still had some limitations. Firstly, the present study only focused on the TGF*β*/smad signaling pathway. Some other pathways may also participate in the treatment of diabetic nephropathy with Tangzhiqing granules, such as Integrin/ILK and WNT/beta-catenin pathways [26]. Further researches should focus on more different pathways to elucidate the mechanisms of Tangzhiqing granules affecting diabetic nephropathy. Secondly, T2DM was induced by high-fat diet and low-dose STZ as mentioned previously in our research. But using type 2 diabetes genetic model animals such as KK-Ay mice, Zucker diabetic fatty rat, and db/db mice may be better to display the pathology of diabetic nephropathy and minimize the impact of confounding factors. Thirdly, whether Tangzhiqing granules improving DM rats podocyte EMT is related to regulating glucolipid metabolic disorders is still not clear. Further experiments in vitro will be needed to verify the findings.

In conclusion, Tangzhiqing granules could relieve both the structural damage and functional changes significantly of diabetic nephropathy rats. Kidney podocytes EMT existed in diabetic nephropathy rats. Tangzhiqing granules may obviously reverse these podocyte phenotypic changes through affecting TGF*β*/smad signaling pathway. But further researches are still needed to verify the mechanisms.

## Figures and Tables

**Table 1:** Physiological and biochemical parameters in the different groups after 8 weeks of treatment.

Group	BG	BUN	Scr	Upro	KI	CHOL	TG	Weight
	(mmol/L)	(mmol/L)	(*μ*mol/L)	(mg/24 h)	(×10^−3^)	(mmol/L)	(mmol/L)	(g)
Control	8.9 ± 1.6	6.5 ± 1.0	26.6 ± 2.7	69.8 ± 27.8	6.0 ± 0.4	2.0 ± 0.3	0.8 ± 0.2	600.4 ± 43.6
DM	24.9 ± 3.4^*∗*^	17.0 ± 2.4^*∗*^	43.9 ± 4.7^*∗*^	1477.4 ± 193.6^*∗*^	12.4 ± 1.3^*∗*^	3.2 ± 0.6^*∗*^	2.9 ± 1.2^*∗*^	376.1 ± 43.3^*∗*^
TZQ1	22.2 ± 4.2^*∗*^	16.8 ± 1.7^*∗*^	41.0 ± 11.9^*∗*^	1328.9 ± 196.1^*∗*^	11.7 ± 1.2^*∗*^	2.6 ± 0.3^*∗*#^	2.9 ± 1.6^*∗*^	398.1 ± 22.3^*∗*^
TZQ2	19.5 ± 5.9^*∗*#^	10.4 ± 1.5^*∗*#^	24.5 ± 3.5^#^	806.1 ± 361.9^*∗*#^	9.6 ± 0.8^*∗*#^	2.5 ± 0.4^*∗*#^	2.1 ± 1.2^*∗*^	444.3 ± 31.8^*∗*#^
TZQ3	14.0 ± 3.3^*∗*#†^	10.3 ± 1.0^*∗*#^	25.4 ± 2.3^#^	782.4 ± 286.8^*∗*#^	9.5 ± 0.9^*∗*#^	2.3 ± 0.3^#^	1.1 ± 0.2^#^	458.5 ± 18.7^*∗*#^

Data were means ± SD; ^*∗*^*P* < 0.05 versus Control group; ^#^*P* < 0.05 versus DM group; ^†^*P* < 0.05 versus TZQ2.

## References

[1] Roselli S., Heidet L., Sich M. (2004). Early glomerular filtration defect and severe renal disease in podocin-deficient mice. *Molecular and Cellular Biology*.

[2] Yamaguchi Y., Iwano M., Suzuki D. (2009). Epithelial-mesenchymal transition as a potential explanation for podocyte depletion in diabetic nephropathy. *American Journal of Kidney Diseases*.

[3] Zhang M., Liu M., Xiong M., Gong J., Tan X. (2012). Schisandra chinensis fruit extract attenuates albuminuria and protects podocyte integrity in a mouse model of streptozotocin-induced diabetic nephropathy. *Journal of Ethnopharmacology*.

[4] Lv Z., Hu M., Zhen J., Lin J., Wang Q., Wang R. (2013). Rac1/PAK1 signaling promotes epithelial-mesenchymal transition of podocytes in vitro via triggering *β*-catenin transcriptional activity under high glucose conditions. *International Journal of Biochemistry and Cell Biology*.

[5] Gaikwad A. B., Viswanad B., Ramarao P. (2007). PPAR*γ* agonists partially restores hyperglycemia induced aggravation of vascular dysfunction to angiotensin II in thoracic aorta isolated from rats with insulin resistance. *Pharmacological Research*.

[6] Gupta J., Gaikwad A. B., Tikoo K. (2010). Hepatic expression profiling shows involvement of PKC epsilon, DGK eta, Tnfaip, and Rho kinase in type 2 diabetic nephropathy rats. *Journal of Cellular Biochemistry*.

[7] White K. E., Bilous R. W. (2000). Type 2 diabetic patients with nephropathy show structural—functional relationships that are similar to type 1 disease. *Journal of the American Society of Nephrology*.

[8] Zhou J., Sun W., Yoshitomi H. (2015). Qiwei granules alleviates podocyte lesion in kidney of diabetic KK-Ay mice. *BMC Complementary and Alternative Medicine*.

[9] Welsh G. I., Coward R. J. M. (2010). Podocytes, glucose and insulin. *Current Opinion in Nephrology and Hypertension*.

[10] Liu Y. (2010). New insights into epithelial-mesenchymal transition in kidney fibrosis. *Journal of the American Society of Nephrology*.

[11] Kang Y. S., Li Y., Dai C., Kiss L. P., Wu C., Liu Y. (2010). Inhibition of integrin-linked kinase blocks podocyte epithelial-mesenchymal transition and ameliorates proteinuria. *Kidney International*.

[12] Jim B., Ghanta M., Qipo A. (2012). Dysregulated nephrin in diabetic nephropathy of type 2 diabetes: a cross sectional study. *PLoS ONE*.

[13] Gerke P., Sellin L., Kretz O. (2005). NEPH2 is located at the glomerular slit diaphragm, interacts with nephrin and is cleaved from podocytes by metalloproteinases. *Journal of the American Society of Nephrology*.

[14] Koop K., Eikmans M., Baelde H. J. (2003). Expression of podocyte-associated molecules in acquired human kidney diseases. *Journal of the American Society of Nephrology*.

[15] Shaw A. S., Miner J. H. (2001). CD2-associated protein and the kidney. *Current Opinion in Nephrology and Hypertension*.

[16] Salant D. J., Topham P. S. (2003). Role of nephrin in proteinuric renal diseases. *Springer Seminars in Immunopathology*.

[17] Li Y., Kang Y. S., Dai C., Kiss L. P., Wen X., Liu Y. (2008). Epithelial-to-mesenchymal transition is a potential pathway leading to podocyte dysfunction and proteinuria. *The American Journal of Pathology*.

[18] Zhang C., Hu J.-J., Xia M. (2010). Protection of podocytes from hyperhomocysteinemia-induced injury by deletion of the gp91phox gene. *Free Radical Biology and Medicine*.

[19] Jinde K., Nikolic-Paterson D. J., Huang X. R. (2001). Tubular phenotypic change in progressive tubulointerstitial fibrosis in human glomerulonephritis. *American Journal of Kidney Diseases*.

[20] Yu D., Petermann A., Kunter U., Rong S., Shankland S. J., Floege J. (2005). Urinary podocyte loss is a more specific marker of ongoing glomerular damage than proteinuria. *Journal of the American Society of Nephrology*.

[21] Fu X., Song B., Tian G.-W., Li J.-L. (2014). The effects of the water-extraction of *Astragali Radix* and *Lycopi herba* on the pathway of TGF-smads-UPP in a rat model of diabetic nephropathy. *Pharmacognosy Magazine*.

[22] Cai H., Liu F., Zuo P. (2015). Practical application of antidiabetic efficacy of *Lycium barbarum* polysaccharide in patients with type 2 diabetes. *Medicinal Chemistry*.

[23] Chen H., Feng R., Guo Y., Sun L., Jiang J. (2001). Hypoglycemic effects of aqueous extract of Rhizoma Polygonati Odorati in mice and rats. *Journal of Ethnopharmacology*.

[24] Wu Q. H., Chen S. Y. (1991). Screening research for Chinese medicine treatment of diabetes with single drug and effective prescriptions. *Journal of Guangzhou University of Chinese Medicine*.

[25] Fang X.-K., Gao Y., Yang H.-Y. (2008). Alleviating effects of active fraction of Euonymus alatus abundant in flavonoids on diabetic mice. *American Journal of Chinese Medicine*.

[26] Dai C., Stolz D. B., Kiss L. P., Monga S. P., Holzman L. B., Liu Y. (2009). Wnt/*β*-catenin signaling promotes podocyte dysfunction and albuminuria. *Journal of the American Society of Nephrology*.

